# The effectiveness of multiparametric magnetic resonance imaging in bladder cancer (Vesical Imaging-Reporting and Data System): A systematic review

**DOI:** 10.1080/2090598X.2020.1733818

**Published:** 2020-03-01

**Authors:** Roberto Carando, Luca Afferi, Giancarlo Marra, Wojciech Krajewski, Vincenzo Pagliarulo, Mohammad Abufaraj, Evanguelos Xylinas, Xavier Cathelineau, Rafael Sanchez-Salas, Marco Moschini

**Affiliations:** a Department of Urology, Clinica Luganese Moncucco, Lugano, Switzerland; b Clinica S. Anna, Swiss Medical Group, Sorengo, Switzerland; c Ward of Surgery and Urology, Clinica S. Chiara, Locarno, Switzerland; d Department of Urology, Luzerner Kantonsspital, Spitalstrasse, Luzern, Switzerland; e Division of Urology, Department of Surgical Sciences, University of Studies of Torino, Turin, Italy; f Department of Urology and Oncologic Urology, Wrocław Medical University, Wroclaw, Poland; g Department of Urology, University “Aldo Moro”, Bari, Italy; h Division of Urology, Department of Special Surgery, The University of Jordan, Amman, Jordan; i Department of Urology Bichat Hospital, Paris Descartes University, Paris, France; j Department of Urology, Institut Mutualiste Montsouris and Université Paris Descartes, Paris, France

**Keywords:** Radical cystectomy, urothelial carcinoma, VI-RADS, muscle-invasive bladder cancer

## Abstract

**Objective:**

To evaluate the role of the Vesical Imaging-Reporting and Data System (VI-RADS) score in the diagnostic pathway of bladder cancer.

**Methods:**

A systemic search of the contemporary literature was performed in December 2019 using the Medical Literature Analysis and Retrieval System Online (MEDLINE), the Excerpta Medica dataBASE (EMBASE), and Web of Science databases focussing on all available articles on VI-RADS.

**Results:**

Overall, six of 15 articles were included. All the available articles evaluated the ability of radiologists to use the VI-RADS score for discriminating non-muscle-invasive bladder cancer (NMIBC) from muscle-invasive bladder cancer (MIBC). Considering a cut-off VI-RADS score of >2, the sensitivity, specificity, positive (PPV) and negative predictive value (NPV) were 78–91.9%, 85–91%.1, 69–78%, and 88–97.1%, respectively. Considering a VI-RADS score cut-off of >3, the sensitivity, specificity, PPV and NPV were 77–94.6%, 43.9–96.5%, 51.6–86%, and 63.7–93%, respectively. Good interobserver agreement was demonstrated in the evaluated studies with a κ score of 0.73–0.89. Only one study evaluated the utility of VI-RADS in determining the presence of MIBC in patients treated with transurethral resection of the bladder diagnosed with high-grade T1 before the second transurethral resection using a VI-RADS score cut-off of >2; the sensitivity, specificity, PPV and NPV were 85%, 93.6%, 74.5%, and 96.6%, respectively.

**Conclusion:**

The VI-RADS score, using multiparametric magnetic resonance imaging, showed excellent results in discriminating MIBC from NMIBC. Preliminary results have been reported for its use in patients with high-grade T1 bladder cancer. These results need to be validated in high-quality real-world settings.

**Abbreviations:**

DCE: dynamic contrast enhancement; DWI: diffusion-weighted imaging; (N)MIBC: (non-)muscle-invasive bladder cancer; mpMRI: multiparametric MRI; TURBT: transurethral resection of bladder tumour; (N)(P)PV: (negative) (positive) predictive value; SC: structural category; T2W: T2-weighted; VI-RADS: vesical imaging-reporting and data system

## Introduction

Multiparametric MRI (mpMRI) has been widely adopted in diagnosing and risk-stratifying prostate cancer [1], and shown promising results in differentiating cystic renal masses [2]. Recently, its use has been proposed in the diagnostic pathway of bladder cancer with the development of the Vesical Imaging-Reporting and Data System (VI-RADS) score [3].

Clinical management of bladder cancer is currently determined mainly based on clinical T Stage determination using transurethral resection of bladder tumour (TURBT) as a diagnostic and therapeutic surgical tool [4]. By reporting tumour invasion at the time of TURBT, urologists are able to decide whether to proceed with radical cystectomy, a morbid procedure associated with impaired quality of life [5–7], or in the case of non-muscle-invasive bladder cancer (NMIBC) to treat the tumour with less invasive methods, such as TURBT followed by adjuvant intravesical instillation. In this regard, TURBT is not a perfect diagnostic and therapeutic tool, with an up to 50% residual tumour rate and 10% progression rate diagnosed at re-TURBT [8]. These rates depend on surgical experience and tumour characteristics.

The use of mpMRI has been proposed to increase the preoperative diagnostic ability in differentiating NMIBC and MIBC to improve the effectiveness of TURBT. The aim of the present systematic review was to summarise the current evidence on the VI-RADS, and its possible implications and future directions in the management of patients with bladder cancer.

## Methods

**Figure 1.**
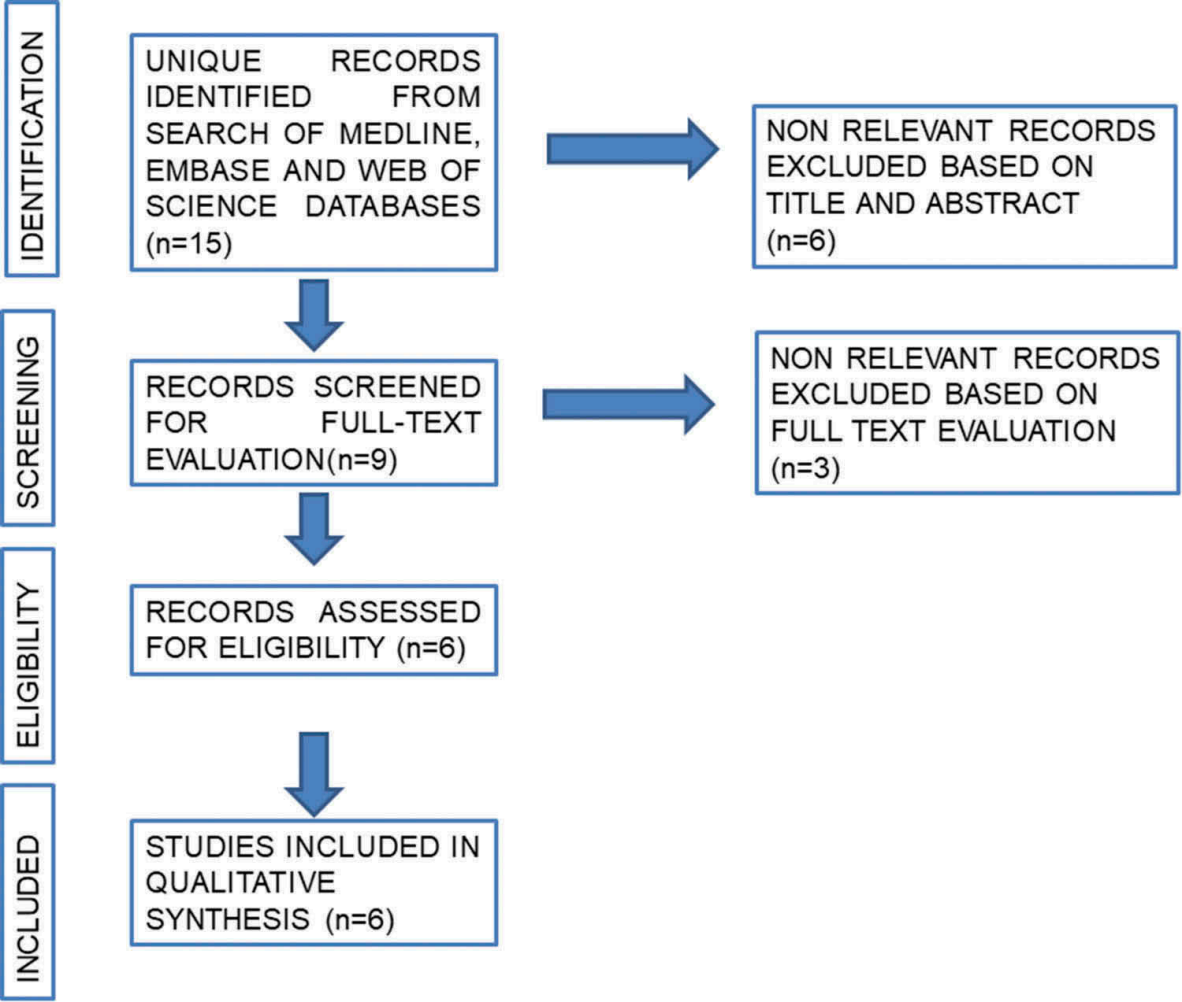
Flow diagram of the search results.

A systematic literature review was performed in December 2019 using the Medical Literature Analysis and Retrieval System Online (MEDLINE), the Excerpta Medica dataBASE (EMBASE), and Web of Science databases. Review articles, editorials and congress abstracts were excluded. Search terms included ‘VI-RADS’ in combination or alone with the terms ‘radical cystectomy’ OR ‘bladder cancer’ OR ‘transurethral resection’. The search was limited to the English literature. References cited in selected articles and in review articles retrieved in our search were also used to identify manuscripts that were not included in the initial searches. The articles that provided the highest level of evidence were then evaluated, where existing prospective studies were preferred to retrospective designs. A list of articles judged to be highly relevant by the first and senior authors was circulated amongst the co-authors and a final consensus was reached on the structure of the review and the articles included. The systematic review was performed in agreement with the Preferred Reporting Items for Systematic Reviews and Meta-Analyses (PRISMA) guidelines [9] (Figure 1).

### The development of VI-RADS

Although several articles exists supporting the role of mpMRI in patients with bladder cancer [10,11], the first description of the VI-RADS score was performed by Panebianco et al. [3] in 2018. A multidisciplinary team composed of members from Europe, North America, South America and Asia with a Delphi-like consensus protocol proposed a 5-point score. The risk of muscle invasion was to be assessed using T2-weighted (T2W) MRI, diffusion-weighted imaging (DWI), and dynamic contrast enhancement (DCE). Considering patient selection and preparation, the authors’ recognised that previous TURBT and intravesical instillation might cause oedema of the bladder wall, which can be confused with tumour invasion, resulting in overestimation of local stage. Therefore, similarly to prostate cancer (with regards to prostate biopsies), MRI examination should be performed before or ≥2 weeks after TURBT or intravesical instillation [12]. Additionally, gas collections (bubbles) in the bladder can cause distortion of the imaging and therefore a 3-day interval between cystoscopy or removal of a catheter and MRI is recommended. The patient should be prepared with the use of an antispasmodic agent to minimise artefacts from bowel peristalsis [13]. The bladder should be distended, and patients should be instructed to not void 1–2 h before imaging or to start drinking 500–1000 mL water in the 30 min before the examination. No distention, as well as overdistension, might lead to a misdiagnosis or over staging of any tumours that are present. Optimally, the bladder should contain ~300 mL at the time of the MRI [14].

From a technical point of view, MR equipment of 1.5 or 3.0 T is recommended to achieve high spatial resolution and signal-to-noise ratio. The T2W image, DWI and DCE MRI are key components of the mpMRI examination. All images should include the whole bladder, proximal urethra, pelvic nodes and prostate if the patient is a male. In females, uterus, ovaries, fallopian tubes and vagina should be included. A spin-echo T1-weighted image can be used in cases of haemorrhage and clots in the bladder or bone metastases. A detailed technical explanation can be found in the original article from Panebianco et al. [3].

The 5-point VI-RADS score is finally created using the individual structural category (SC), diffusion-weighted (DW) category, and contrast-enhanced (CE) category from T2W, DWI and DCE MRI to predict the probability of muscle invasion. With a VI-RADS 1 score, muscle invasion is highly unlikely: SC, CE and DW category 1. With a VI-RADS 2 score, muscle invasion is unlikely to be present: SC, CE and DW category 2, both CE and DW category 2 with SC category 3. VI-RADS 3 score, presence of muscle invasion is equivocal: SC, CE and DW category 3, or SC category 3 CE or DW category 3, the remaining sequence category 2. VI-RADS 4 score, muscle invasion is likely: at least SC and/or DW and CE category 4, the remaining category 3 or 4; SC category 3 plus DW and/or CE category 4; SC category 5 plus DW and or CE category 4. VI-RADS 5 score, invasion of muscle and beyond the bladder is very likely: at least SC plus DW and/or CE category 5, the remaining category 4 or 5.

## Results

### The effectiveness of mpMRI in differentiating NMIBC from MIBC

An overview of the published studies evaluating the ability of VI-RADS to discriminate NMIBC from MIBC is presented in Table 1 [15–20]. Differentiating NMIBC and MIBC is crucial for counselling patients with bladder cancer. Surgical management of NMIBC and MIBC is completely different and using mpMRI to differentiate it might reduce costs and perioperative complications. Different results were obtained considering different VI-RADS cut-offs, when considering a cut-off VI-RADS score of 2, sensitivity, specificity, positive (PPV) and negative predictive value (NPV) were 78–91.9%, 85–91.1%, 69–78%, and 88–97.1%, respectively. Considering a cut-off of VI-RADS >3, sensitivity, specificity, PPV and NPV were 77–94.6%, 43.9–96.5%, 51.6–86%, and 63.7–93%, respectively. Considering a cut-off of VI-RADS >4, sensitivity, specificity, PPV and NPV were 76–91%, 76–93%, 83.3%, and 78.9%, respectively.

**Table 1.** Studies reporting results of VI-RADS.

Reference	Patients, *n*	Years of accrual	Aim	Sensitivity, %	Specificity, %	PPV, %	NPV, %	AUC	Number of observers, inter-observer agreement
Barchetti et al. 2019 [19]	75	2017–2018	Evaluate accuracy and inter observer variability using VI-RADS for discrimination between NMIBC and MIBC	Reader 1: 91 (VI-RADS >2) Reader 2 82 (VI-RADS >2) Reader 1: 82 (VI-RADS >3) Reader 2 77 (VI-RADS >3)	Reader 1: 89 (VI-RADS >2) Reader 2 85 (VI-RADS >2) Reader 1: 94 (VI-RADS >3) Reader 2 89 (VI-RADS >3)	Reader 1: 77 (VI-RADS>2) Reader 2 69 (VI-RADS>2) Reader 1: 86 (VI-RADS>3) Reader 2 74 (VI-RADS>3)	Reader 1: 96 (VI-RADS >2) Reader 2 92 (VI-RADS >2) Reader 1: 93 (VI-RADS >3) Reader 2 91 (VI-RADS >3)	Reader 1: 0.93 Reader 2: 0.87	2, Inter-observer agreement for the overall score: κ = 0.731
Del Giudice et al. 2019 [15]	231	2017–2019	1. Differentiation between NMIBC and MIBC 2. Avoiding second TUR after TUR in H-R NMIBC	Reader 1 91.9 (VI-RADS >2) Reader 2: 85 (VI-RADS >2)	Reader 1: 91.1 (VI-RADS >2) Reader 2: 93.6 (VI-RADS >2)	Reader 1: 77.5 (VI-RADS >2) Reader 2: 74.5 (VI-RADS >2)	Reader 1: 97.1 (VI-RADS >2) Reader 2: 96.6 (VI-RADS >2)	Reader 1. 0.94 Reader 2. 0.93	2, κ = 0.81
Makboul et al. 2019 [16]	50	n.r.	Differentiation between NMIBC and MIBC	78 (VI-RADS >2)	88 (VI-RADS >2)	78 (VI-RADS >2)	88 (VI-RADS >2)		2, κ = 0.87
Kim et al. 2019 [17]	297	2015–2019	Differentiation between NMIBC and MIBC	91.3 (VI-RADS >4) 94.6 (VI-RADS >3)	76 (VI-RADS >4) 43.9 (VI-RADS >3)	83.3(VI-RADS >4) 51.6 (VI-RADS >3)	78.9(VI-RADS >4) 63.7 (VI-RADS >3)	n.r.	2, (κ = 0.89 for T2 W, κ = 0.82 for DWI, and κ = 0.85 for DCE
Wang et al. 2019 [18]	340	2011–2018	Differentiation between NMIBC and MIBC	87.1 (VI-RADS >3)	96.5 (VI-RADS >3)	n.r.	n.r.	0.94	n.r.
Ueno et al. 2019 [20]	74	2010–2018	Differentiation between NMIBC and MIBC	76 (VI-RADS >4) 88 (VI-RADS >3)	93 (VI-RADS >4) 77 (VI-RADS >3)	n.r.	n.r.	0.90	5, Interclass correlation coefficient: 0.85

AUC: area under the curve; H-R: high-risk; n.r.: not reported.

Most published studies had more than one radiologist included and inter-observer agreement analyses were performed with κ statistics, in order to evaluate the variability of radiologists’ evaluation of mpMRI with the VI-RADS score. Overall, good inter-observer agreement was found with a κ score ranging between 0.73 and 0.89. To date, Del Giudice et al. [15] is the only prospective evaluation, they used a VI-RADS score cut-off of 1–2 vs 2–5 to differentiate superficial from invasive disease at TURBT. Further data are required to validate these findings in real-world settings.

### The effectiveness of mpMRI in avoiding second TURBT in high-risk NMIBC

Recently, aside from the classical differentiation of NMIBC from MIBC, a new application of the VI-RADS score has been proposed by Del Giudice et al. [15]. They propose its application for avoiding a second TURBT after a first TURBT, where a high-risk NMIBC was diagnosed. In fact, current guidelines recommend a second TURBT in patients diagnosed with high-risk NMIBC or in cases of T1 bladder cancer without muscle in the specimen [4]. Repeating TURBT in these patients allows urologists to find patients where a muscle invasion was misdiagnosed or to find residual NMIBC tumours [8]. However, this procedure is related to high costs, risk of complications and could potentially be avoided in a high percentage of patients where no further tumour would be diagnosed. Del Giudice et al. [15] selected 114 patients with high-risk NMIBC before second TURBT and evaluated the VI-RADS system in diagnosing residual MIBC. They found a sensitivity, specificity, PPV and NPV of 85%, 93.6%, 74.5% and 96.6%, respectively. These results support the utility of VI-RADS in the selection of candidates for a second TURBT; however, still no data support its role in diagnosing residual NMIBC tumours.

### Potential limitations of the VI-RADS score

Although these preliminary results (Table 1) showed that the VI-RADS score can play an important role in the diagnostic pathway of patients with bladder cancer, still some limitations need to be highlighted and discussed. First, inter-observer disagreement has been reported in the existing literature [15–19]. There is an urgent need to evaluate the VI-RADS score in real-world settings to ensure that even in non-tertiary referral centres, this system can offer the same results as those described by experienced radiologists. Second, some pathological parameters cannot be assessed by imaging such as carcinoma *in situ*, lymphovascular invasion, and the presence of histological variants, which play an important role in determining the optimal therapeutic and prognostic management of patients with bladder cancer [21–23]. Third, the new molecular classification of the bladder cancer [24], although still not clinically validated has already shown promising results in determining the necessity of chemotherapy, radiation therapy or immunotherapy. Still, the presence of an accurate pathological evaluation is still necessary for individualised therapy [25]. Therefore, MRI staging based on the VI-RADS classification may be useful in daily practice but does not give an overview of the tumour’s biology.

## Conclusion

The VI-RADS score showed excellent results in discriminating MIBC from NMIBC. Preliminary results confirmed similar findings in patients diagnosed with high-grade T1 NMIBC candidates for a second TURBT. Although some limitation exists, such as the absence of pathological specimen for evaluation of morphological and genomic prognostic factors, the VI-RADS score seems a promising tool in the diagnostic pathway of bladder cancer.
